# Notes and News

**Published:** 1905-12-16

**Authors:** 


### Notes and News.

A National Costume Ball, to be held in the Empress Rooms on January 30, is being organised by Lady Maud B. Wilbraham in aid of the West Ham Hospital.

A lady who does not desire her name to be known has offered to give £1,000 towards the £3,000 debt to the bankers, incurred by the Royal Hospital for Incurables, Putney, if the other £2,000 be obtained by the beginning of the new year.

On Monday, December 18, a Variety Entertainment will take place at the Alexandra Theatre, Stoke Newington, N. Mr. H. G. Dudley Bennett will give the entire proceeds in aid of the Metropolitan Hospital, Kingsland Road, N.

A special appeal is being made on behalf of University College Hospital to meet the expenses entailed by the completion of the new building and the opening of eighty-six additional beds. The Committee have already been obliged to sell stock to the extent of about £5,000 to provide funds for their immediate needs.

Mr. Roosevelt has appointed a commission of three surgeons-general, representing the army, navy, and marine hospital services, to inquire into the conditions prevailing in Government workshops and offices, and to recommend measures for the prevention of tuberculosis among Government employés in these places.

The Poor-law Commission met for the first time on Tuesday afternoon at the Foreign Office, Lord George Hamilton taking the chair. All the Commissioners were present, and general arrangements were made for the business meetings which are to follow. It was decided not to admit the representatives of the Press to the deliberations of the Commission, but the question of issuing periodical statements for publication was left open. We regret the determination of the Commissioners because we believe that the publication of the reports of their proceedings from time to time would be of considerable educational value to the public.

The Grocers' Company have made a grant of £50 to the Clergy Nursing Home in Nottingham Place for the building of the new hostel.

Under the will of Sir John Groves, of Rodwell, near Weymouth, bequests of £100 each have been left to the Dorset County Hospital and the Weymouth Hospital.

At the invitation of Princess Christian and Princess Victoria of Schleswig-Holstein, a meeting was held on November 8 at Schomberg House, Pall Mall, in support of the work of the League of Mercy in the districts of Berkshire, Buckinghamshire, Hampshire, and Oxfordshire, of which Princess Victoria is Lady President. The report of the work for the year, which was read by the Organising Secretary, Mr. Reginald Lund, stated that the Princess started the League in Berkshire at the request of the Prince of Wales, the Grand President, in 1904. The result was that in that year her Highness was enabled to give £773 to the League, and in addition, £202 to the Royal Berkshire Hospital, being half the proceeds of a fancy fair held at Buckhurst, Wokingham. The Prince of Wales granted another sum of £100 to the hospital, making the amount received by it during 1904 over £300. Owing to the successful working of the League in Berkshire, extension of territory has been granted to her Highness by the Prince of Wales. At the present moment Princess Victoria has 53 lady Vice-Presidents collecting under her, and the number is steadily increasing. The amount which will be received by the League of Mercy this year from the four districts will exceed £1,000.

The award of the Nobel Prizes took place on the 10th instant in the banqueting hall of the newly-built Nobel Institute, at Stockholm. Although the British Empire has no representative among the recipients this year, there is not likely to be any feeling other than the warmest approval in regard to the manner in which the candidates for this very high honour have been selected. The prize for physics is awarded to Professor Philip Lenard, of Kiel, chiefly on account of his work on cathode rays. The prize for chemistry goes to Professor Adolf von Bäyer, of Munich, well known in connection with the chemistry of dyes. The Polish writer, Henryk Sienkiewiez, receives the prize for literature. The peace prize is awarded to an Austrian lady, Baroness Bertha von Suttner, President of the Austrian Peace Society. In medicine, the prize is given to Dr. Robert Koch for his work and discoveries in connection with tuberculosis. The award to Dr. Koch will certainly give much satisfaction to the members of the medical profession, who are able to appreciate the great advantages which Dr. Koch's life work has bestowed upon suffering humanity. His discovery of the bacillus of tuberculosis is, of course, his greatest achievement, and is that with which his name is chiefly associated. But his labours extended, with considerable profit to mankind, into many other fields of pathology; and no more worthy recipient could have been found on whom to bestow the Nobel Prize in Medicine.

